# Functional Outcome of Arthroscopic Anterior Cruciate Ligament Reconstruction Using a Quadrupled Hamstring Autograft: A Prospective Study

**DOI:** 10.7759/cureus.96654

**Published:** 2025-11-12

**Authors:** Suvank Rout, Rishab Garg, Ronish Patidar, Souvagya Rout

**Affiliations:** 1 Medicine, Topiwala National Medical College and B.Y.L. Nair Charitable Hospital, Mumbai, IND; 2 Trauma and Orthopaedics, Colchester General Hospital, Colchester, GBR; 3 Orthopaedics, Baroda Medical College and Sir Sayajirao General (SSG) Hospital, Baroda, IND; 4 Orthopaedics, Topiwala National Medical College and B.Y.L. Nair Charitable Hospital, Mumbai, IND; 5 Orthopaedics, Indira Gandhi Medical College, Shimla, IND; 6 Radiology, First Faculty of Medicine, Charles University, Prague, CZE

**Keywords:** acl tear, anterior cruciate ligament (acl) reconstruction, hamstring autografts, knee, proprioception knee

## Abstract

Background

The anterior cruciate ligament (ACL) is one of the primary stabilisers of the knee, and injury to it often requires surgical intervention. ACL injuries are common sports injuries that tend to occur during sudden deceleration, pivoting, or landing movements, resulting in knee instability and decreased functionality. Different graft options have been proposed over the years for reconstructing the ACL.

Objective

This study aimed to evaluate the short-term functional, clinical stability, and biomechanical (muscle strength) outcomes following arthroscopic ACL reconstruction using a quadrupled semitendinosus and gracilis autograft performed in a single tertiary care centre.

Methodology

We conducted a prospective study involving 40 patients undergoing primary ACL reconstruction at a tertiary care centre in India. The study was conducted from March 2022 to May 2024. The surgical technique involved harvesting and quadrupling the hamstring (semitendinosus and gracilis) tendons, with arthroscopic fixation using an adjustable-loop device on the femur and a bioabsorbable interference screw on the tibia. All patients followed an accelerated rehabilitation protocol. The outcomes were assessed at a mean follow-up of 14.2 months using the Lysholm Knee Scoring Scale, isokinetic dynamometry, and clinical stability tests.

Results

The results were positive and demonstrated a significant improvement in knee function (p < 0.001). The mean Lysholm score increased from approximately 49 preoperatively to 95 at the final follow-up. Ninety percent of patients achieved an excellent outcome, and the remaining 10% achieved a good outcome. All patients demonstrated normal knee stability on clinical testing. Ninety-five per cent returned to their pre-injury level of work or sports nine months postoperatively. The most common presenting complaint was the knee "giving way" or instability of the knee joint. While 40% of patients reported mild, transient anterior knee pain, there were no cases of graft failure, infection, or other serious complications.

Conclusion

Arthroscopic ACL reconstruction with a quadrupled hamstring tendon autograft provides excellent short-term outcomes and is a reliable technique for restoring knee stability and function. Further studies are required to evaluate objective outcomes and long-term sequelae.

## Introduction

The anterior cruciate ligament (ACL) is the primary stabiliser of the knee joint, preventing excessive anterior translation of the tibia over the femur [1]. ACL ruptures are among the most common major knee injuries worldwide, with an annual incidence of between 60 and 70 per 100,000 persons [2,3]. ACL injuries often occur during sudden deceleration, pivoting, or landing movements, commonly seen in American football, soccer, or basketball [3]. Rupture of the ACL results in knee instability and abnormal joint kinematics, increasing the risk of meniscal injuries and articular cartilage damage, thereby predisposing the joint to early osteoarthritis [4].

In young, active patients, surgical reconstruction of the ACL is generally regarded as the treatment of choice, as it restores stability and often permits patients to return to pre-injury activities. Arthroscopic reconstruction of the ACL is now considered the standard approach, as it allows more accurate and anatomic graft placement through small incisions. This approach minimises trauma to the extensor mechanism and allows rapid rehabilitation, resulting in lower morbidity [5,6].

Various graft types are available for ACL reconstruction, the most common being bone-patellar tendon-bone (BPTB) grafts and hamstring tendon (semitendinosus-gracilis) grafts [7]. It has often been noted that the graft selection depends on the surgeon's expertise, preference, tissue availability, and priority. The BPTB grafts, which are harvested from the central third of the patellar tendon, are noted to have high strength but have been documented to cause anterior knee pain, patellar tendon shortening, loss of terminal extension of the knee joint range of motion, loss of extensor strength, and patellofemoral arthritis [8,9]. In comparison, hamstring grafts have very high tensile strength and avoid patellar morbidity, leading to their increasing popularity for ACL reconstruction. While BPTB graft preparation is standardised, different suturing and preparation techniques for hamstring tendon grafts, such as the quadrupled hamstring graft, four-strand graft, tripled semitendinosus graft, and semitendinosus quadruplication, have been proposed in recent years [10]. The quadrupled semitendinosus and gracilis graft, in particular, is theorised to offer superior tensile strength and stiffness compared with doubled preparations, potentially enhancing initial stability and promoting earlier rehabilitation. However, comprehensive studies assessing the combination of patient-reported function, clinical stability, and objective muscle strength recovery for this specific graft configuration remain limited. Much of the existing literature groups different hamstring techniques together, potentially obscuring the unique outcomes of the quadrupled construct. Although the main drawbacks of hamstring autografts include early loss of knee flexion strength and potential tunnel widening, these issues have been mitigated by modern fixation techniques [10].

Modern ACL reconstruction utilises arthroscopy with robust fixation devices (e.g., adjustable-loop cortical buttons and interference screws). Combined with structured rehabilitation, this approach has been shown to reduce complications and improve clinical and functional outcomes [5,6]. The goals of ACL reconstruction are to restore a stable, painless knee and enable the patient to return to pre-injury sports or occupational activities. In this study, we evaluated functional outcomes after arthroscopic ACL reconstruction using a quadrupled semitendinosus-gracilis autograft.

## Materials and methods

Study design and patient selection

We performed a prospective study of patients who underwent primary ACL reconstruction at a tertiary care centre between March 2022 and May 2024. Institutional Ethics Committee for Biomedical and Health Research (IECBHR), Baroda Medical College and Sir Sayajirao General (SSG), Baroda, issued approval (IECBHR/166-2022).

Inclusion and Exclusion Criteria 

In our study, we included patients aged 18 to 50 years who were diagnosed with a complete ACL tear, confirmed clinically and by MRI. Patients with multi-ligamentous injuries, advanced osteoarthritis, intra-articular fractures, associated bleeding or coagulation disorders, or prior knee surgeries were excluded from this study. Immunocompromised patients and those unwilling to provide consent were also excluded. Patient demographics and injury mechanisms were recorded.

Preoperative Workup

Patients diagnosed clinically and radiologically were admitted and underwent routine investigations such as full blood counts (FBC), urea and electrolytes (U&Es), chest radiographs, and electrocardiograms (ECGs). The patients also underwent anaesthetic assessment for regional or general anaesthesia.

Demographic and injury data were recorded: side of injury, mechanism (categorised as road traffic accident, sports injury, or fall), occupation (sedentary, athletic, or labourer), symptoms, and time from injury to surgery. Preoperative knee stability was assessed in the clinic (Lachman, anterior drawer, and pivot-shift tests) and reassessed under anaesthesia during surgery [11-16]. Patients were also educated on static and dynamic quadriceps exercises whilst awaiting surgery. All tests and scoring systems used are open access, and no licence is required.

Surgical method

All patients undergoing single-bundle ACL reconstruction were positioned supine, with the uninvolved limb supported. Prophylactic antibiotics, typically ceftriaxone, were administered before tourniquet inflation, and the limb was elevated to facilitate exsanguination. The patients first underwent diagnostic arthroscopy through an anterolateral port to examine all knee compartments and address associated pathologies such as meniscal tears. An oblique incision was made medial to the tibial tuberosity to harvest the gracilis and semitendinosus tendons. These tendons were cleaned, whipstitched, looped into a quadrupled graft, sized, and pretensioned on a graft masterboard.

The arthroscope was introduced into the joint cavity via the anterolateral portal, and a shaver was introduced through the anteromedial portal. The joint was then debrided of ligamentum plicae, fat pads, and synovial reflections to enhance visibility of the ACL footprint and the lateral femoral condyle. Care was taken to avoid damaging the posterior cruciate ligament (PCL). The torn ACL was then visualised, typically presenting as a scarred stump attached to the PCL, the intercondylar notch roof, or absent from its femoral attachment. The residual ACL tissues were excised, preserving the remnants at the tibial and femoral attachment sites. The femoral remnant acted as a landmark for positioning the guide pins for the femoral tunnel, while the tibial remnants were preserved to serve as a potentially neurologically active envelope for the graft, which may aid proprioceptive function. The intercondylar notch of the femur was then shaped and enlarged (notchplasty) to clear the native footprint and prevent future graft impingement.

An anatomical single-bundle reconstruction was then performed by drilling a femoral tunnel via the anteromedial portal and a corresponding tibial tunnel. The prepared graft was pulled through the tibial tunnel into the femoral tunnel and secured with an endobutton. The graft was then cyclically tensioned (20-30 times) before being fixed on the tibial side with an interference screw. Finally, the wounds were closed, the knee was braced, and a standardised accelerated rehabilitation protocol was initiated immediately, supported by postoperative antibiotics and wound care. Weight-bearing was allowed with crutches as tolerated. Physical therapy emphasised closed-chain exercises, progressive strength training, and neuromuscular control.

The accelerated ACL physical therapy protocol delineated a six-week programme. From weeks 0 to 2, the primary rehabilitation goals were to achieve full knee extension, attain 90 degrees of knee flexion, develop adequate quadriceps strength, and establish a normal gait pattern. By weeks 2 to 4, the target range of motion should progress to 0-120 degrees, with full weight-bearing permitted on crutches and an absence of limping. Between weeks 4 and 10, patients should advance toward a full range of motion by approximately week 6, incorporating lunges and progressive strengthening of lower extremity muscle groups through the complete hamstring and quadriceps range. From weeks 12 to 16, rehabilitation should emphasise continued flexibility training, further quadriceps strengthening, and the development of sport-specific cardiovascular endurance. From 3 months onwards, plyometric exercises and jogging should be initiated, provided quadriceps strength has reached at least 65% and the knee demonstrates a full range of motion and stability. From months 5 to 6, the focus shifts toward sport-specific drills and agility training. Return to sport is generally appropriate at approximately 6 to 9 months postoperatively, which depends on achieving knee flexion greater than 130 degrees, hamstring strength exceeding 90%, quadriceps strength exceeding 85%, and successful completion of sport-specific agility activities.

Outcome measures and follow-up

Patients were evaluated at 3, 6, and 12 months after surgery. At the final follow-up (mean 14.2 ± 3.7 months), knee stability tests were repeated. Knee laxity was evaluated using the Lachman, anterior drawer, and pivot-shift tests. Comparing the laxity of both knees yielded the side-to-side difference (involved knee versus uninvolved knee), which served as an indicator of restored knee stability in the reconstructed knee. Functional outcome was measured using the Lysholm Knee Scoring Scale [11-16].

For quadriceps and hamstring strength analysis, the peak extension and flexion torque were isokinetically measured using the PrimusRS (BTE Technologies, Hanover, Maryland) instrumented dynamometer at 60 deg/sec (for strength; average value of 5 repetitions) and 240 deg/sec (for muscle endurance; average value of 15 repetitions) [17]. The strength index (the ratio of the peak torque of the involved leg to the uninvolved leg, multiplied by 100) was used as the representative parameter for thigh muscle strength. The endurance index was similarly calculated. This allowed us to analyse any change in muscle strength and endurance following surgery, and to determine when muscle strength and endurance had recovered. Paired Student’s t-test was used to compare the results between the two muscle groups statistically.

The Lysholm Knee Scoring Scale is a patient-reported outcome measure used to evaluate the functional status of the knee joint, consisting of eight questions assessing a patient’s knee function related to pain, swelling, giving way, locking, limping, stair climbing, squatting, and support. Each question is scored on a scale from 0 to 10, with a total possible score of 100. Scores were categorised as excellent (>90), good (84-90), fair (65-83), or poor (<65). We also recorded any complications, graft laxity or retear, and the patient’s ability to return to pre-injury occupation or sports. Descriptive statistics were calculated and represented as means or percentages [15].

Data and statistical analysis

The data were analysed using IBM SPSS Statistics for Windows, Version 29 (Released 2022; IBM Corp., Armonk, New York). Preoperative and postoperative continuous variables, such as Lysholm Knee Scores and quadriceps and hamstring strength indices, were compared using the Student’s t-test. For t-tests, we reported the t-statistic as well as the degrees of freedom (df). For categorical variables, such as complication rates and return to pre-injury level of activity, comparisons were assessed using the chi-square (χ²) test or Fisher’s exact test, as appropriate. For chi-square tests, the χ² value and degrees of freedom were reported along with the p-value. Effect sizes were calculated using Cohen’s d for paired t-tests and Cramér’s V for chi-square tests. All statistical tests were two-tailed, with significance set at p < 0.05, and 95% confidence intervals (CIs) were reported for odds ratios (ORs), where appropriate. This ensured complete documentation and transparency for all inferential statistical analyses.

## Results

Patient demographics and injury characteristics

After exclusion, we identified 40 patients who met our study criteria. Of these, 28 patients (70%) were in the 21-30-year age group, with a mean age of 29.4 years. The cohort showed a male predominance, with 33 patients (82.5%). Injuries were nearly evenly distributed between the right knee (52.5%) and the left knee (47.5%). Road traffic accidents (30 patients, 75%) were the most common mechanism of injury. Seven patients (17.5%) sustained ACL tears due to sports activities, and three patients (7.5%) sustained ACL tears due to falls or slips. The majority of patients (27 patients, 67.5%) had sedentary jobs, while seven (17.5%) were amateur athletes and six (15%) performed manual labour.

At the time of presentation, all patients reported a sense of instability (“giving way”) and difficulty descending stairs. Knee pain was present in 62.5% of patients, swelling in 30%, locking in 22.5%, and difficulty walking on uneven surfaces in 60%. The time from injury to surgery was ≤6 weeks in 35% of patients, 2-4 months in 40%, 4-6 months in 15%, and >6 months in 10%.

Preoperative findings

Preoperatively, nine patients showed associated knee injuries on MRI, with seven having medial meniscus tears and two having lateral meniscus tears. No collateral or PCL injuries were observed. In the clinic, the majority of patients had a positive Lachman test (95%), a positive anterior drawer test (90%), and a positive pivot shift (55%). Under anaesthesia at surgery, knee laxity was even more pronounced, with all patients (100%) showing positive Lachman and anterior drawer tests, and the majority showing a positive pivot shift (95%).

Surgical details and complications

The average femoral tunnel length in our study was 37.1 mm (range, 31-40 mm), and the average tibial tunnel length was 38.4 mm (range, 31-40 mm). Graft diameters averaged 8-9 mm. Fixation was achieved with an Endobutton on the femur and an interference screw on the tibia in all cases (Tables 1, 2). No intraoperative complications were noted. At the final follow-up, postoperative anterior knee pain, reported by 16 patients (40%), was the most common complication. This pain was generally mild and localised to the graft harvest site. There were no cases of surgical site infection, fixed flexion deformity, extension deficit, graft laxity, or neurovascular injury. No patient required revision or reoperation during follow-up.

**Table 1:** Femoral Tunnel Length

Femoral Tunnel Length (in mm)	Number of Patients
31-32	01
33-34	07
35-36	09
37-38	16
39-40	07

**Table 2:** Tibial Tunnel Length

Tibial Tunnel Length (in mm)	Number of Patients
31-32	01
33-34	05
35-36	02
37-38	13
39-40	19

Functional outcomes 

Twenty-three patients (57.5%) attended follow-up for 13-16 months, and 11 patients (27.5%) were followed up for 8-12 months. By the final follow-up, 36 patients (90%) had an "excellent" outcome and 4 patients (10%) had a "good" outcome according to the Lysholm criteria; no patient was rated "fair" or "poor." This represented a marked improvement, as 35 patients had "poor" scores and 5 patients had "fair" scores preoperatively, whereas postoperatively, 4 patients had "good" outcomes and 36 patients had "excellent" outcomes at the final follow-up. This change was statistically significant (paired t-test, p < 0.001), reflecting recovery of knee function. All patients achieved a full range of motion, and knee stability tests were normalised postoperatively (negative Lachman test and anterior drawer test in all patients) (Table 3). The preoperative mean Lysholm score was 48.6 ± 7.2, and the postoperative mean was 95.4 ± 3.8. The paired t-test for the Lysholm knee score showed a significant improvement from preoperative to postoperative values, with t(39) = 32.6, p < 0.001, and a very large effect size (Cohen’s d = 5.16).

**Table 3:** Comparison of Preoperative and Postoperative Lysholm Score

Lysholm Score	Preoperative (n)	Postoperative (n)
Poor (<65)	35	00
Fair (65–83)	05	00
Good (84-90)	00	04
Excellent (>90)	00	36
Total	40	40

Thirty-eight patients (95%) returned to their pre-injury level of work or activity by the time of the final follow-up. Two patients (5%), both with "good" Lysholm scores, did not return to their previous occupation due to residual pain or personal fear of reinjury.

Postoperative evaluation demonstrated a significant improvement in both quadriceps and hamstring isometric strength indices compared with preoperative values. At six months postoperatively, the isometric strength of the quadriceps improved from 62.8 ± 8% preoperatively to a postoperative mean of 91 ± 5%, with t(39) = 15.8, p < 0.001, and Cohen’s d = 2.5. Similarly, hamstring strength increased from a preoperative mean of 64 ± 7% to a postoperative mean of 90 ± 6%, with t(39) = 14.3, p < 0.001, and Cohen’s d = 2.3. Comparison between the operated and contralateral normal limb showed no statistically significant difference in quadriceps strength (t(39) = 1.2, p = 0.23) or hamstring strength (t(39) = 1.1, p = 0.28), indicating near-complete restoration of muscle function. Additionally, quadriceps power improved significantly following surgery (p = 0.002), achieving values comparable to the unaffected limb. Hamstring power (p = 0.001) similarly demonstrated improvement without a significant interlimb difference, suggesting balanced muscle recovery. Direct comparison of postoperative quadriceps and hamstring strength indices revealed no statistically significant difference (p = 0.214), indicating symmetrical recovery of flexor and extensor muscles and appropriate rehabilitation balance.

All seven patients who sustained sports-related injuries regained sufficient knee function to return to competitive play. These athletes had excellent final Lysholm scores (>95) and resumed sport within 9-12 months.

## Discussion

Our prospective study evaluates the functional and muscle performance outcomes following ACL single-bundle reconstruction using quadrupled hamstring autografts (semitendinosus and gracilis). Forty patients were assessed for knee stability, muscle strength, and return to activity over a mean follow-up of 14.2 months. We noted a marked improvement in Lysholm scores and clinical stability of the knee joint. Isokinetic testing also showed substantial recovery of muscle strength. Furthermore, 95% of patients successfully returned to their pre-injury level of work or sports, and while 40% reported mild transient anterior knee pain, there were no instances of graft failure, infection, or other serious complications.

Patient demographics and injury characteristics

In our study, the patient population had a mean age of approximately 29 years, with a male predominance. Most ACL injuries were sustained during road traffic accidents or sports, consistent with previous studies showing that young active males performing high-risk activities represent the typical demographic for ACL tears [3,4]. All patients were between 20 and 50 years of age. Twenty-one (52.5%) patients sustained right-sided ACL injuries, while nineteen (47.5%) injured the left side. Arora et al. reported that limb-sidedness does not influence recovery following ACL reconstruction [5].

Thirty-five percent of patients underwent ACL reconstruction within six weeks of injury. Delayed ACL reconstruction is known to increase the incidence of secondary meniscal and cartilage lesions [4]. We observed a meniscal co-injury rate of 22.5%, which is lower than the 50-60% often reported, perhaps reflecting the relatively short delay to surgery in our cohort, with 75% of patients operated on within four months [4].

Graft selection and biomechanical considerations

The primary goal in ACL reconstruction is to restore knee stability [1]. Thus, graft selection in ACL reconstruction is critical, as it remains one of the most easily adjustable factors affecting graft rupture and reoperation rates [8]. ACL reconstruction is predominantly performed as a single-bundle procedure, with autografts being more commonly used than allografts or synthetic grafts. The three most common autograft options are the BPTB graft, the hamstring tendon graft (semitendinosus-gracilis), and the bone-quadriceps tendon (BQT) graft.

BPTB has historically been considered the “gold standard” for ACL reconstruction due to its high strength and stiffness, consistency of the graft size, and ease of harvest [4,10,11]. However, it is associated with donor-site morbidity, including anterior knee pain, patellar tendon shortening, loss of terminal extension, extensor weakness, and patellofemoral arthritis [8,9]. In contrast, hamstring autografts offer comparable mechanical strength with reduced donor-site complications. Their biomechanical properties vary depending on the number and configuration of tendon strands [7]. The reported stiffness values for BPTB grafts range from 158 to 685 N/mm (324-543 N/mm for 10 mm grafts), whereas hamstring grafts report a wider but overlapping stiffness range of 4-1148 N/mm [7]. Strauss et al. [18] demonstrated higher cyclic loading stiffness for hamstring grafts (273 ± 49.5 N/mm) compared with BPTB grafts (151 ± 25.5 N/mm). Similarly, the modulus and ultimate tensile strength of hamstring grafts (145-904 MPa and 66-156 MPa, respectively) are comparable to or higher than those of BPTB grafts (184-338 MPa and 22-101 MPa) [7]. In a meta-analysis of randomised trials, Zhao et al. found no significant difference between BPTB and hamstring grafts in clinical function or stability [19]. Hart et al. found no significant differences in ultimate load to failure between these three commonly used autografts [20]. Given their excellent strength profile and lower risk of anterior knee morbidity, hamstring tendon autografts have become increasingly popular for ACL reconstruction.

Functional and muscle performance outcomes

In our study, the mean Lysholm knee score improved significantly from 48.6 preoperatively to 95.4 postoperatively (paired t-test: t(39) = 32.6, p < 0.001, Cohen’s d = 5.16, 95% CI 44.3-49.1), indicating excellent functional recovery. These results are consistent with Chaudhary et al., Deehan et al., and Jomha et al., who reported postoperative Lysholm scores ranging between 92 and 94 following arthroscopic ACL reconstruction [11,12,21,22]. The excellent-to-good outcomes observed in 100% of our patients further reinforce the reliability of the hamstring autograft as a viable and effective graft choice.

Postoperative evaluation demonstrated a significant improvement in both quadriceps and hamstring isometric strength indices compared to preoperative values. At six months postoperatively, the isometric strength of the quadriceps improved from 62.8 ± 8% preoperatively to 91 ± 5% postoperatively (t(39) = 15.8, p < 0.001, Cohen’s d = 2.5, 95% CI 25-32%), and hamstring strength increased from 64 ± 7% to 90 ± 6% (t(39) = 14.3, p < 0.001, Cohen’s d = 2.3, 95% CI 22-28%). When comparing the operated limb with the contralateral non-operated limb, there was no statistically significant difference for quadriceps (t(39) = 1.2, p = 0.23, 95% CI −1.8 to 3.2) or hamstring strength (t(39) = 1.1, p = 0.28, 95% CI −2.0 to 3.1), indicating near-complete restoration of muscle function. Additionally, there was a significant increase in the power of the quadriceps after surgery (p = 0.002) and hamstrings (p = 0.001), achieving values comparable to the unaffected limb. Direct comparison of postoperative quadriceps and hamstring strength indices revealed no statistically significant difference (t(39) = 1.4, p = 0.21, 95% CI −1.5 to 3.0), indicating symmetrical recovery of flexor and extensor muscles and appropriate rehabilitation balance.

Role of arthroscopy and rehabilitation

Arthroscopic techniques have also likely contributed to rapid recovery. As studies have reported, minimally invasive ACL reconstruction via arthroscopy allows for early motion and strengthening, less postoperative pain, and faster rehabilitation [5,6]. In our practice, patients were mobilised on the first postoperative day, and most regained quadriceps and hamstring strength by six months [23]. According to the ACL rehabilitation protocol by Grinsven et al., to return to sports following ACL surgery, one should have quadriceps strength of at least 80% of the normal leg and hamstring strength of at least 80% of the normal leg [24]. According to Bizzini et al., criteria for returning to sports include hamstring and quadriceps strength of at least 85% compared to the contralateral side and when the patient tolerates sport-specific activities [25]. In our study, hamstring and quadriceps strengths were restored to 91% of the normal leg, suggesting that isometric strengths were restored.

Overall outcomes and clinical relevance

In this series of 40 patients undergoing ACL reconstruction with a quadrupled hamstring tendon graft, we observed excellent functional outcomes in 90% of cases and good results in 10%, consistent with previous literature. Arora et al. reported 88% of patients with good-to-excellent outcomes after hamstring ACL reconstructions [5]. The mean final Lysholm score (approximately 95) in our study compares favourably to reported results (often in the low 90s) for hamstring graft reconstructions [5].

In our study, 95% of the patients could return to their pre-injury occupation levels. This aligns with other studies where successful ACL reconstruction allows a high rate of return to work or sport [9,11]. The outcomes of this study highlight the effectiveness of arthroscopic ACL reconstruction using quadrupled hamstring autografts in restoring knee function and muscle performance. Early postoperative strengthening enhanced graft integration without increasing the risk of graft elongation or failure [25]. Comprehensive rehabilitation was noted to result in balanced recovery of quadriceps and hamstrings, which prevents muscle imbalances and ensures long-term knee stability [26]. Accurate tunnel placement, secure fixation using endobutton and interference screws, and early structured rehabilitation play an important role in overall patient outcomes.

Limitations of the study

Some limitations of our study include the modest sample size (n = 40) and the lack of a control or comparator group (e.g., BPTB graft or other hamstring configurations), which limits the inferential strength of our findings and prevents direct comparative conclusions about the superiority of this specific technique. Secondly, a limited maximum follow-up duration of 24 months (mean of 14.2 months) may result in an inability to capture late graft failure or degenerative changes. Therefore, our positive outcomes should be interpreted as short-term results, and we avoid making claims about long-term performance. Thirdly, we did not employ objective biomechanical testing such as KT-1000 arthrometry to test for knee stability; we used subjective knee stability assessments by comparing to the contralateral leg. We suggest that future studies with larger cohorts, longer follow-up, and inclusion of randomised control comparisons between graft types could provide more substantial evidence.

## Conclusions

In our study, we find that arthroscopic ACL reconstruction using a quadrupled semitendinosus-gracilis tendon autograft yields excellent short-term results. The vast majority of patients achieved a stable, pain-free knee with high Lysholm scores (>90) and returned to their previous level of activity. Donor-site morbidity was low, and no serious complications occurred. These results support the effectiveness of the hamstring autograft in ACL reconstruction, corroborating other studies that report high patient satisfaction and functional success with this technique. Although the comparative effectiveness against other grafts remains to be established, future comparative studies are warranted.
